# The Case of St. Bartholomew's Hospital

**Published:** 1916-08-19

**Authors:** 


### NATIONAL INSURANCE AND THE VOLUNTARY HOSPITALS

### The Case of St. Bartholomew's Hospital.

St. Bartholomew's Hospital took the lead when the National Insurance Act first came into operation, by laying down a course of action in regard to insured persons applying for treatment at the voluntary hospitals, which was practically followed throughout the country. St. Bartholomew's is the senior British hospital, and it is encouraging to find that under Lord Sandhurst, the present treasurer, it is again leading, by pressing upon the Commission of Investigation into the Working of the Insurance Act the extent to which insured persons avail themselves of the benefits conferred by voluntary hospitals.

Lord Sandhurst has dealt only with the experience of St. Bartholomew's Hospital, and in what follows we reprint from his report to the governors for the year 1915 facts and statistics which will be found at the end of this article. They will be studied with interest, and we shall be glad to have similar particulars from the managers of any of the larger voluntary hospitals who will be good enough to forward them for publication.

The matter is one of extreme importance, and the present is an opportunity to drive home the facts that the voluntary hospitals have borne the whole cost of treating injured persons when seriously ill, without any cost to the Government or any payment towards the cost of such treatment. Had the voluntary hospitals declined to give this treatment to injured persons, the insurance scheme of the Government would long ago have been in a state of bankruptcy.

Lord Sandhurst makes some excellent points. He declares it is not too much to claim that, were it not for the services rendered by St. Bartholomew's, in common with other hospitals, in promoting the more rapid restoration to health of the many thousands of insured persons who seek hospital treatment, the demands upon the approved societies in respect of "sickness benefits" would be very seriously increased. Further, it must be remembered that under Section XII., subs. 2 (*c*) of the National Insurance Act, 1911, approved societies were, under certain conditions, empowered to pay to hospitals the whole or part of the "sickness benefit" payable in respect of insured persons being inmates of hospitals, and some hospitals did, in fact, receive a certain though small addition to income thereby.

Upon the amendment of this section in question by the Act of 1913, income from this source practically ceased, the whole of the "benefit" being almost invariably claimed by the insured person. These are the circumstances which have led Lord Sandhurst to express the hope that, recognising the services rendered by the hospitals, each approved society will be disposed to assist St. Bartholomew's and its treasurer in his task of obtaining funds for the adequate maintenance of that institution.

Lord Sandhurst's intervention at the present time is seasonable and important, and we called attention on page 422 of The Hospital of the 5th instant to the fact that the party newspapers have taken certain evidence already given before the Commission of Investigation as vindicating the success of the Insurance Act. We then insisted that to claim that the financial fears of hospital men have not been fulfilled is not correct, seeing that it is the voluntary hospitals and not the Insurance Acts which have provided institutional treatment for the insured, the cost of which has not yet been repaid to the hospitals by the Government. Further, panel practice is a very doubtful boon, and real benefits from the Acts are rather far to seek. We hope, indeed, that Lord Sandhurst's action will lead the larger voluntary hospitals throughout the country to follow suit, and that each of them may approach the approved societies and the Commission of Investigation with the object of making the real facts in regard to these matters known to the Commission, the Government, and also to people of all classes throughout the nation.

### ST. BARTHOLOMEW'S HOSPITAL.

NATIONAL INSURANCE ACT, 1911.

The following figures and facts have been extracted from the treasurer's report for the year 1915 :—

The hospital has now had the experience of three complete years' (1913-14-15) working of the above Act in its effect upon the attendances of persons in the casualty and out-patients' departments. The records for that period are of considerable interest as showing the general uniformity not only of the decrease in the actual numbers of persons applying for treatment, but also the proportion of "insured" to "uninsured" adults treated.

Proportion of Insured to Total In-Patients.

With regard to in-patients, it will be noticed that the proportion of insured persons to the total number of adults admitted has steadily increased. In arriving at the decreases of attendances shown in the following figures comparison is made with the returns for 1912 :—

| | 1913 | 1914 | 1915 |
|---|---|---|---|
| Decrease in total attendances of adults in casualty and out-patients' departments ... ... | 35.3 | 35.0 | 37.5 |
| Proportion of attendances of "insured" persons to total adult attendances ... ... | 31.4 | 33.4 | 34.2 |
| (*a*) Proportion of "insured" males ... ... ... | 43.9 | 45.8 | 43.0 |
| (*b*) Proportion of "insured" females ... ... ... | 16.7 | 22.1 | 22.0 |
| Proportion of "insured" persons to total number of adult persons admitted to the wards ... ... | 50.0 | 54.1 | 56.7 |
| (*a*) Proportion of "insured" males ... ... ... | 66.0 | 68.4 | 73.6 |
| (*b*) Proportion of "insured" females ... ... ... | 33.0 | 40.8 | 42.1 |

*The above return relates to civilian patients only.*

Insured Persons Treated as Out-Patients.

The number of patients referred to the casualty and out-patients' departments by medical practitioners was

6,458. Of this total, 3,291, or 50.9 per cent., were "insured" persons, of whom 2,688, or 40 per cent., were sent up by "panel" doctors.

Total Patients of all Classes.

The following figures show the total number of patients "insured" and "uninsured" treated at the hospital during the three years dealt with above, viz. :—

| | 1913 | 1914 | 1915 |
|---|---|---|---|
| In-patients ... ... ... | 8,984 | 8,948 | 8,648 |
| Casualty and out-patients (first attendances) | 79,887 | 76,974 | 68,425 |
| Subsequent attendances ... | 196,077 | 200,243 | 193,439 |
| Total attendances ... ... | 275,964 | 277,217 | 261,864 |

